# Hidden GBV: Women and substance use

**DOI:** 10.3389/fpsyt.2022.939105

**Published:** 2022-08-08

**Authors:** Eilidh Moir, Sophie Gwyther, Heather Wilkins, Gillian Boland

**Affiliations:** ^1^NHS Tayside, Dundee, United Kingdom; ^2^Dundee Health and Social Care Partnership, Dundee, United Kingdom; ^3^Dundee Volunteer and Voluntary Action, Dundee, United Kingdom; ^4^Dundee Women's Aid, Dundee, United Kingdom

**Keywords:** women, substance and alcohol use, GBV, support service needs, recovery, gendered services

## Abstract

Gender based violence (GBV) is disproportionately higher in women who use substances. This vulnerable population are also at a disadvantage when it comes to accessing harm reduction services. Between May and October 2021 there were 77 cases discussed at Multi-Agency Risk Assessment Conferences (MARAC) in Dundee. The majority of these cases (62) had substance misuse as a risk factor. It is at these meetings that the vulnerability of women comes to the fore and issues of violence are highlighted. During this time period, 44 cases involved the victim being strangled/choked or suffocated and 43 cases had weapons as a risk factor. 56 of the cases included children. The issue of GBV or Intimate Partner Violence (IPV) is often hidden, especially in and by those affected by substance use. Women experiencing GBV require specialist support, often across different services to address different needs. How well violence against women is understood in relationships that are affected by drugs is difficult to determine. In many instances it isn't until a MARAC meeting, or similar crisis point is met, that the extent of the abuse is highlighted. What services can provide singularly is limited in many of these cases but joint-working and innovative practices, such as the Hub model and the Gendered Services Project in Dundee, strive to change the landscape of service delivery.

## Introduction

The issue of substance misuse across Scotland has long been a cause for concern, particularly in the city of Dundee. Situated in Eastern Scotland, and a third of the local authorities that make up the region of Tayside, Dundee is most commonly cited as the drug death capital of Europe (1). The deaths figures for Dundee are stark and numbers in the city continued to rise between 2016 and 2020 to equate to a rate of 43.1 per 100,000 of the population (2). In 2019 deaths from drugs in Dundee increased by 8%, the breakdown of this rise by sex proved to be even more alarming. Deaths in men fell by 2% during this year but deaths in women increased by 37%.

In 2018, already with a lens on rising deaths from drugs, the Scottish Government published findings to try to understand what was already a disproportionate increase in deaths in women. The report cited multiple interconnected factors as rationale for such a stark rise in the number of deaths among women who use drugs. These include co-morbidities, polypharmacy and aging, but also bereavement and loss of maternal or parenting roles. Crucially, what the report highlighted was what the authors termed, “ongoing risk among women engaged with drug treatment services, potentially reflecting failures to meet needs or missed opportunities” [3, p. 9]. Although service provision for those who use substances was available, the needs of women, and indeed engaging women in services, were not being met.

Dundee city had the highest police recordings of domestic abuse in Scotland in 2020–2021, with 177 per 10,000 of the population (Scotland figure is 119 per 10,000 population) (3)^1^. Further, sexual crimes in Dundee and across Scotland rose in the same year; For Dundee this was an increase of 45% and an overall increase of 15% for Scotland compared to the previous year (4).

Secrecy around violence is common and often not visible until a crisis point is met (5). For some, this might be presentation at a service who then raise significant concerns, enough to call for a Multi-Agency Risk Assessment Conference (MARAC) to be held. As an example, within a 6-month period in Dundee (May to October 2021), 77 MARAC meetings were held. The vast majority of these cases discussed substance misuse alongside weapons and violence as risk factors. Further, 56 cases acknowledged that children were involved or at risk.

MARACs are convened in order for specialists across voluntary and statutory sectors, including an Independent Domestic Violence Advisor (IDVA), to share information they have about a victim. The assumption of the MARAC is that no one agency will know the full details of the victim's life and experiences but they can all add perspective and evidence that are important to the victim's safety. The outcome for each meeting is that a plan for safety and support for the victim is made by the specialists around the table. Recommendations for safeguarding children and managing the behavior of the perpetrator can also be made. It is the strength of interagency working and the distinct knowledge and expertise of each agent which enables cross working and positive outcomes to be achieved. It is reported that up to 60% of domestic abuse victims report no further violence following IDVA support and MARAC interventions (6).

If we know that drug deaths in women are rising in Dundee against a backdrop of violence in terms of domestic and sexual abuse, can services recognize and respond to these complex needs? Local evidence gathered from women accessing substance misuse services uncovers the complexities of drug use, shining a light on the pivotal roles that GBV and mental ill health have in increasing vulnerability in women. It is posited here that substance misuse organizations and homelessness shelters respond to the gendered expectations of men and women, not the complex vulnerabilities they actually experience. More men than women tend to access services and women have specific needs and barriers in place which can make access more difficult for them.

Taking a gendered approach means looking at the group who are having difficulty accessing services (women) and redesigning those services to meet their needs more effectively. Gaining insight from lived experience is crucial to this to recognize, for example, barriers for access to services, trauma, stigma, and GBV. The outcome for services can then be a better understanding of, in this case women's needs, and creating change in the way they deliver support.

## Substance use and GBV

Literature on substance use and violence surrounding women is scarce and has tended to focus on alcohol use owing to a smaller and harder to reach drug use population (7). Therefore, gathering sufficient data to understand the scope of the problem continues. A link between perpetration of physical violence and substance use in men has been discussed but there are limited studies into the relationship between women's substance use and experiences (8).

In Canada, studies estimate that anywhere between 25 and 50% of women who are engaged with substance use treatment programs have experienced violence (9). Moreover, substance use specialists working in the treatment facilities report they are ill-equipped to support women experiencing violence alongside substance use, similar to the findings noted above for Dundee and Scotland. The literature describes services that provide support for issues such as substance use and mental health as the product of language, funding, and models. The services themselves then evolve discretely around specific themes or sources of finance. Services focusing on one issue can be at the exclusion of others and it is in the lack of addressing the co-existence of issues, such as substance use and GBV, and the vulnerabilities that emerge from substance use, that gaps in service provision can be seen (10).

The problem of hidden GBV in a female population who use substances may be larger than we currently understand. Studies in England and Scotland show that women may avoid accessing services due to stigma, concerns about losing children, shame, or previous negative interactions (11). As a result, they and their experiences may not appear in research data, and if they do, the data is so small that no insight or conclusions can be drawn (11, 12). This lack of data, perhaps due to women's fears of disclosing information or embarrassment, means that what we know about women's experiences, and complex needs they present, is likely to be the tip of the iceberg.

## Understanding complex need

Interagency and more specifically, engaging specialists to come together to find solutions and put in place support and interventions, appears to have an impact on complex cases involving substance use and violence. Women's services in Dundee have been working to try to understand and learn from this type of cross-agency working. Dundee Women's Aid (DWA) became increasingly concerned a number of years ago about a group of women affected by domestic abuse issues. At their presentation to DWA refuge services women described a range of other complex needs, including substance use. A group of local organizations led by DWA undertook a test of change to explore how interventions were coordinated and delivered within existing resources. The test of change allowed DWA to secure funding to develop a project whereby women with multiple complex needs could access urgent psychological assessment, treatment planning, and intervention. Moreover, this work led to crucial conversations within local strategic planning groups. These conversations explored gender and the specific needs that women may have that might prevent them from accessing appropriate care and support, and where they do engage with services, engagement being successful.

A study was carried out by researchers at the University of Dundee to explore these themes and capture need. Focus groups with 39 women who use services and 53 members of staff were conducted (13). The findings showed that women felt that services did not take them or their concerns seriously, and in some cases even judging or placing conditions on access to support. Further, it was reported that staff were not trained in GBV and as a result did not know how to respond adequately to women seeking help (13).

## Service redesign

There are then, two cross cutting themes around women's substance use and GBV: Getting women into services and redesigning services to better suit women's needs. In many ways these two themes are interdependent. The current, albeit small, body of research tells us that there's an imperative to remove the vacuum of addressing substance misuse for women as a singularity and instead integrating other services not only to attract women into recovery but also meet their needs (14).

The context of treatment services, both statutory and third sector, in the UK sits within a changing landscape of budgetary constraints. For statutory services, various changes to health, social and treatment services have meant under-staffing, under-skilled staffing, lack of continuity, cuts in provision or all out closure of services (15). Although there is a rhetoric of recovery-oriented care, wider determinants such as changes to the benefits system affect women in particular. Looking more closely at existing services, embedding practices of creating trauma informed environments have been explored in research into women and homelessness (16). The core recommendations from the research echo much of the findings of the Dundee and Scotland research and reports, referenced earlier: the creation of female-specific support and services, training and supporting staff to respond to trauma and women's complex needs, link with specialists in GBV to increase knowledge and best support those in services, and to guarantee equitable access for all women [(16) p. 21, (17)].

It is clear that there is alignment amongst those investigating service improvements for women, particularly in the spheres of substance use and GBV. We know that in a lot of cases, a crisis threshold is met before violence is understood and addressed. However, there are also barriers to accessing services that are particular to women. Evidence shows that a large proportion of women do not access services and in instances where they do engage, it is short-lived and ineffectual (13, 18). Further anecdotal local evidence on women's disengagement with services, investigated by Tayside Council on Alcohol, identified reading and writing difficulties, limited access to forms, documents and bank accounts, lack of access to the internet and stigma and safety fears.

## Future direction: The “Hub” model

The recognition that women's issues are distinct and a drive toward women-specific service design permeates the current conversation. What we understand about need in Dundee from local services, and figures on domestic abuse and sexual crimes, is that women's needs are complex and there is an imperative for service approaches to adapt to these. The Dundee Drugs Commission in 2019, convened to investigate the impact of drug use and the response of services in the city, called for gender-sensitive approaches to service planning [(19), p. 6]. Service users interviewed by the Commission highlighted gender-specific need and an appetite for recognising and responding to gendered experiences (15). Individualised services do go some way in recognising need, irrespective of gender but the literature and experiences of service users suggests more can be done. Specifically, in training staff to be confident and knowledgeable in responding to gender- based violence, allowing timely support which fits into women's lives around work or childcare and in improving the co-ordination of multi-agency responses to women with complex needs.

In Dundee there has been a move toward improving services for women through the Gendered Services Project. The Project engages women with lived experience to influence its direction and to ensure that the challenges and needs of women with complex needs are represented. So far, women involved in the project have presented many of the themes discussed here: barriers to accessing services, stigma especially in relation to substance use and gender-based violence, the lack of a trauma informed approach within services, and a lack of trusting relationships. It is the work of the Project to drive these issues forward to inform and engage local services and create change. To be effectual and embed this thinking into services for women, Women's Rape and Sexual Abuse Centre is the lead partner in a bid to create Dundee's first Women's Hub. In partnership with Dundee Drug and Alcohol Recovery Service, Criminal Justice Service Women's Team, Tayside Council on Alcohol and third sector partners, We Are With You, Hillcrest Futures, Dundee Women's Aid and Barnardos, the Women's Hub will be a one stop shop for women with substance use and complex needs. The Hub will allow direct access to support from a range of organisations using a joined-up approach to service provision. Services will be trauma informed and person centred. This model could be replicated in other areas to improve the quality of relationships between services, really address the complex issues women experience, provide a holistic, emotionally and physically safe space and work from a strengths-based empowerment model where resilience is emphasised over issues and problematic behaviour. The challenge services face in supporting women who have complex substance use and GBV issues is considerable but there are strategies, such as The Hub model, that aim to provide gendered, joined-up approaches of support and empowerment.

## Data availability statement

The original contributions presented in the study are included in the article/supplementary material, further inquiries can be directed to the corresponding author.

## Author contributions

SG contributed to writing, provided data and local Dundee research. HW provided wider literature and local data. GB provided local data and context. EM contributed to literature search, called writing meetings, structured the paper, and contributed to writing. All authors contributed to the article and approved the submitted version.

## Funding

This publication was funded from the Gendered Services Project by the Corra Challenge Fund (Ref No - Challenge Fund-19/972).

## Conflict of interest

The authors declare that the research was conducted in the absence of any commercial or financial relationships that could be construed as a potential conflict of interest.

## Publisher's note

All claims expressed in this article are solely those of the authors and do not necessarily represent those of their affiliated organizations, or those of the publisher, the editors and the reviewers. Any product that may be evaluated in this article, or claim that may be made by its manufacturer, is not guaranteed or endorsed by the publisher.

## Footnotes

^1^Across Scotland in 2020-2021, the highest incidence rate was among the age group 31-35 years old for both victims and accused. Nine in ten incidents happened inside the home and four in five incidents involved a female victim and male accused.
